# Hepatoprotective Effect of Mitochondria-Targeted Antioxidant Mito-TEMPO against Lipopolysaccharide-Induced Liver Injury in Mouse

**DOI:** 10.1155/2022/6394199

**Published:** 2022-06-20

**Authors:** Peng-Fei Wang, Ke Xie, Yun-Xing Cao, An Zhang

**Affiliations:** Department of Critical Care Medicine, The Second Affiliated Hospital of Chongqing Medical University, Chongqing, China

## Abstract

The liver is vulnerable to sepsis, and sepsis-induced liver injury is closely associated with poor survival of sepsis patients. Studies have found that the overproduction of reactive oxygen species (ROS) is the major cause of oxidative stress, which is the main pathogenic factor for the progression of septic liver injury. The mitochondria are a major source of ROS. Mito-TEMPO is a mitochondria-specific superoxide scavenger. The aim of this study was to investigate the effect of Mito-TEMPO on lipopolysaccharide- (LPS-) induced sepsis mice. We found that Mito-TEMPO pretreatment inhibited inflammation, attenuated LPS-induced liver injury, and enhanced the antioxidative capability in septic mice, as evidenced by the decreased MDA content and the increased SOD activity. In addition, Mito-TEMPO restored mitochondrial size and improved mitochondrial function. Finally, we found that the levels of pyroptosis-related proteins in the liver of LPS-treated mice were lower after pretreatment with Mito-TEMPO. The mechanisms could be related to Mito-TEMPO enhanced antioxidative capability and improved mitochondrial function, which reflects the ability to neutralize ROS.

## 1. Introduction

Sepsis is a systemic disease elicited by an unregulated host reaction to infection [1], which is the primary cause of mortality in the intensive care unit and imposes a great challenge for clinicians with restricted therapeutic options [2]. The liver plays an important role in immune homeostasis and energy metabolism, and it is vulnerable in the acute phase of sepsis [3]. Septic liver injury, as a common clinical feature, is regarded as an independent indicator of mortality [4]. In addition, liver dysfunction is strongly associated with poor survival of sepsis patients; damaged hepatocytes could release damage-associated molecular patterns to trigger systemic inflammatory responses, exacerbating the dysfunction of the liver and other organs [5]. Therefore, effective protection of liver function is crucial for the treatment of sepsis and the improvement of patient prognosis.

Sepsis is characterized by hyperactivation of the immune response, which triggers an excessive inflammatory response [6]. Recent studies have demonstrated that oxidative stress is a common mechanism of sepsis-induced liver injury. It not only directly leads to genotoxic damage (DNA damage) but also exacerbates the inflammatory pathway to amplify the injury of hepatocytes [7]. Oxidative stress is regarded as an imbalance between reactive oxygen species (ROS) production and elimination by antioxidant systems [8]. In fact, inflammation and oxidative stress are inseparably interconnected. Inflammation is the initial response in the early stage of sepsis and it induces the production of ROS, and the generation of ROS results in oxidative stress [9]. Excessive ROS can also trigger the inflammatory cascade and amplify the inflammatory response via the release of proinflammatory factors, such as IL-6 and TNF-*α* in hepatocytes, resulting in liver damage [10]. ROS and inflammation have mutual stimulatory effects and form a vicious feedback loop, which causes sustained damage [11]. Recent work in this field suggests that ROS contribute to Nod-like receptor 3 (NLRP3) inflammasome activation [12, 13]. It is well known that activation of the NLRP3 inflammasome plays a major role in pyroptosis, which is an inflammation-dependent type of programmed cell death [14]. Studies have indicated that pyroptosis is related to various types of disease, including Alzheimer's disease, acute liver injury, and acute kidney injury [15]. Hence, we can deduce that the accumulation and overproduction of excessive ROS exacerbate pyroptosis, aggravating acute liver injury. Thus, eliminating ROS or reducing ROS production may be a potential strategy for sepsis-induced liver injury therapy. In fact, the protection of organs from excessive ROS by antioxidants is an attractive area in cancer chemoprevention [16]. However, the role of antioxidants in sepsis-induced liver injury is still unclear.

The mitochondrion is an important organelle for cell energy metabolism, which modulates the redox status, cell growth, and death [17]. As a major site of ROS generation, mitochondria have received considerable attention. It was recently discovered that mitochondrial ROS (mtROS) directly stimulate the production of proinflammatory cytokines and contribute to the development of disease, such as autoimmune diseases and cardiovascular diseases [18]. Of note, mitochondria are also a predominant target of ROS, which can easily lead to mitochondrial dysfunction [19], which is closely related to sepsis-induced organ dysfunction [20]. Therefore, in the last few years, mitochondria-targeted antioxidant therapeutics have been proposed as novel therapies for inflammatory diseases and cancers [21]. Recent studies have indicated that the main challenge of mitochondria-targeted antioxidant therapy is to maintain adequate concentrations of antioxidants at ROS-producing sites [22]. Mito-TEMPO is a mitochondria-targeted antioxidant with strong antioxidant activity and a specific scavenger of mitochondrial superoxide. Studies have found that Mito-TEMPO can accumulate severalfold within mitochondria [23]. Its antioxidative property has been extensively verified in various diseases. A recent study found that Mito-TEMPO rescued burn-induced cardiac dysfunction by recovering cardiac mitochondrial dysfunction [24]. In addition, one study suggests that Mito-TEMPO alleviates aldosterone-induced renal tubular cell injury by inhibiting apoptosis [25]. However, the role of Mito-TEMPO in sepsis-induced acute liver injury remains uncertain. The purpose of this study was to investigate the protective effect of Mito-TEMPO against sepsis-induced liver injury. In addition, the influence of Mito-TEMPO on oxidative stress and pyroptosis was further explored.

## 2. Materials and Methods

### 2.1. Animal Experiments

Male 8-week-old C57BL/6 mice were supplied by Chongqing Medical University. Prior to the experiments, the mice were housed in a specific pathogen-free environment for at least 1 week with free access to food and water. The care and use of mice were approved by the Chongqing Medical University Institutional Animal Care and Use Committee. The experimental protocol was approved by the Ethics Committee of the Second Affiliated Hospital of Chongqing Medical University (lot number: 2020-21).

### 2.2. LPS-Induced Sepsis Mouse Model and Mito-TEMPO Pretreatment

To investigate the role of Mito-TEMPO in sepsis-induced liver damage, a lipopolysaccharide- (LPS-) induced experimental sepsis mouse model was established by injecting male mice intraperitoneally with LPS (*E. coli* 0111: B4, Sigma, USA) at a dosage of 5 mg/kg body weight. The control group was injected with the same volume of sterile phosphate-buffered saline (PBS), as in previous studies [26, 27].

The mice were randomized and divided into three groups: the control group, the LPS group, and the LPS+Mito-TEMPO group (pretreatment with Mito-TEMPO followed by LPS injection). Mito-TEMPO (Sigma, USA) was intraperitoneally injected at a dose of 20 mg/kg body weight 1 h prior to LPS injection. The dosage of Mito-TEMPO is comparable with previous studies [28, 29]. Animals were euthanized 24 h after LPS administration, and whole blood and liver samples were harvested for analysis.

### 2.3. Cytokine Measurement

Whole blood was harvested and serum was prepared as previously described [30]. Liver tissues of septic mice were homogenized with PBS (10 mg tissues/100 *μ*l of PBS), and the supernatants of homogenates were collected according to the manufacturer's instructions. Serum alanine transaminase (ALT) and aspartate transaminase (AST) levels were measured using commercial ALT and AST assay kits (Jiancheng, Nanjing, China) to reflect liver injury. Increased serum levels of inflammatory cytokines are critical pathological factors and valuable indicators of sepsis [31]. The levels of inflammatory factors including IL-6, IL-10, IL-1*β*, and TNF-*α* in serum and liver homogenates were determined with ELISA kits (eBioscience, USA) to indicate systemic inflammation.

### 2.4. Histological Analysis

Liver tissues of septic mice were harvested and fixed in 4% paraformaldehyde overnight at 4°C and embedded in paraffin. Next, 4 *μ*m thick slices were placed on glass. The slices were stained with H&E to assess histological damages using light microscopy. The histopathologic scoring analysis was conducted in a blinded manner using previously described methods [32, 33]. In brief, the following six indicators were scored: vacuolization, nuclear condensation, nuclear fragmentation, nuclear fading, erythrocyte stasis, and inflammatory cell infiltration. Scores were assigned based on the percentage of cells showing these phenomena in five microscopic fields (×200) as follows: 0 = 0%, 1 = 0–10%, 2 = 10–50%, and 3 = 50–100%. The six subscores were then summed; the higher the total score, the more severe the injury.

### 2.5. TUNEL Staining

TUNEL staining was conducted as described in a previous study [34]. In brief, liver tissues of septic mice were fixed, embedded, and sectioned into 4 *μ*m thick slices. The Apoptosis Assay Kit (Beyotime) was used to detect apoptotic cells in liver tissue sections according to the manufacturer's instruction. The liver slices were deparaffinized, rehydrated with graded ethanol dilutions, incubated with 20 *μ*g/ml Proteinase K (Beyotime) at 37°C for 30 min, and washed with PBS three times. Then, the slices were incubated with 50 *μ*l TUNEL working solution at 37°C for 60 min, followed by incubation with DAPI (Beyotime) for 15 min protected from light. The slices were washed with PBS three times, and the fluorescence signals (488 nm excitation wavelength) were observed by fluorescence microscopy. For statistical analysis, three fields under ×200 magnification were observed and TUNEL-positive cells were counted, as previously described [35].

### 2.6. Immunofluorescence Staining

Liver paraffin slices were deparaffinized and rehydrated. Tris-ethylenediaminetetraacetic acid (EDTA) retrieval solution (Servicebio, China) was used for antigen retrieval, and then, 5% BSA was used to incubate the slices. Then, slices were incubated with anti-mouse caspase-1 (1 : 1000, Servicebio) or anti-mouse HSP60 (1 : 3000, Servicebio) overnight at 4°C in a humidified chamber. The slices were subsequently incubated with PE-Cy3-conjugated secondary antibody (for caspase-1) or FITC-conjugated secondary antibody (Servicebio, Wuhan, China; for HSP60) at room temperature in the dark. DAPI was used to stain the nuclei. Images were obtained by a confocal microscope (Nikon), and statistical analysis was performed using Image-Pro Plus 6.0 software as described in a previous study [36].

### 2.7. Western Blot

The livers of septic mice were collected and lysed with RIPA buffer. Total and nuclear proteins were extracted using the RIPA Lysis Buffer (Beyotime, Beijing, China) and a nuclear extraction kit (Beyotime, Beijing, China), respectively. Protein samples were separated using 10–12% SDS-PAGE and transferred onto PVDF membranes. Then, the membranes were washed and blocked for 120 min, followed by incubation with primary antibodies against NF-*κ*B p-p65 (Abcam, USA), histone H3 (Affinity, China), Sirt3 (Abcam, USA), PGC-1*α* (Abcam, USA), SOD2 (Abcam, USA), acetylated SOD2 (Ac-SOD2) (Abcam, USA), caspase-1 (Abcam, USA), GSDMD (Abcam, USA), IL-1*α* (Abcam, USA), IL-1*β* (Abcam, USA), or GAPDH (Affinity, China) at 4°C overnight. All antibodies were diluted according to the manufacturers' instructions. The membranes were washed and incubated with secondary antibodies at room temperature for 1 h. Finally, the blots were detected using enhanced chemiluminescence substrate (ECL kit, Millipore). Signal intensities were measured using Fusion software.

### 2.8. RNA Extraction and Quantification

Quantitative real-time PCR (qPCR) was performed as described previously [37]. Briefly, total mRNA was extracted from the liver samples of septic mice using RNAiso plus reagent (Takara Bio, China). cDNA was synthesized using the Takara cDNA synthesis kit (Takara Bio, China) according to the instruction manual. qPCR was performed, and the mRNA levels of the genes encoding NF-*κ*B, PGC-1*α*, caspase-1, Gasdermin-D (GSDMD) were measured using TB Green™–Premix Ex Taq™ II (Takara, China) on a CFX96 system (Bio-Rad CFX96, CA, USA). The primers were synthesized by Tsingke-biology (Chongqing, China). The primer sequences are listed in what follows: NF-*κ*B: sense 5′-CGACGTATTGCTGTGCCTTC-3′, antisense 5′-TGAGATCTGCCCAGGTGGTAA-3′; caspase-1: sense 5′-GAAACGCCATGGCTGACAAG-3′, antisense 5′-CGTGCCTTGTCCATAGCAGT-3′; PGC-1*α*: sense 5′-AAAGGGCCAAGCAGAGAGA-3′, antisense 5′-GTAAATCACACGGCGCTCTT-3′; GSDMD: sense 5′-GCGATCTCATTCCGGTGGACAG-3′, antisense 5′-TTCCCATCGACGACATCAGAGAC-3′; GAPDH: sense 5′-TTCACCACCATGGAGAAGGC-3′, antisense 5′-GGCATGGACTGTGGTCATGA-3′. The 2^−*ΔΔ*Ct^ method was used to assess the mRNA levels.

### 2.9. Oxidative Stress Assays

Malondialdehyde (MDA) and superoxide dismutase (SOD) are oxidative stress-associated indicators [38]. MDA levels in the serum of septic mice were measured with the Lipid Peroxidation MDA Assay Kit (Beyotime, S0131M) according to the manufacturer's instructions, as described in a previous study [39]. In brief, thiobarbituric acid (TBA) working solution and standard curves were prepared. Then, 100 *μ*l serum was mixed with the TBA working solution, and the mixture was heated by boiling water for 15 min, cooled down to room temperature, and centrifuged at 1000 *g* for 10 min. About 0.2 ml of the supernatant was collected, transferred to a 96-well plate, and measured with a microplate reader (Bio-Rad Instruments) at 532 nm.

The SOD levels in serum were detected by the Total Superoxide Assay Kit (Beyotime, S0101M) according to the manufacturers' instructions, as previously described [40, 41]. In brief, working solution and SOD standards were prepared. Then, 20 *μ*l serum or SOD standards were mixed with working solution in a 96-well plate. The mixture was incubated at 37°C for 30 min and measured with a microplate reader (Bio-Rad Instruments) at 450 nm. The SOD standard curve was made and used for all samples.

### 2.10. Liver mtROS Assays

mtROS levels in liver tissues of septic mice were measured by MitoSOX™ Red reagent (Thermo Fisher, USA) as described in a previous study [42, 43]. Briefly, 5 *μ*M MitoSOX™ reagent working solution was prepared. The fresh liver tissues were frozen and cut by a cryostat into 5 *μ*m thick slices. Then, the slices were incubated with MitoSOX dye for 30 min at 37°C. DAPI was used for nuclear detection. The images were taken with a fluorescence microscope (excitation/emission maxima: 510/580 nm). MitoSOX fluorescence intensity was quantified using Image-Pro Plus software.

### 2.11. Electron Microscopic Assessment

Fresh mouse liver tissues were fixed in 2.5% glutaraldehyde, dehydrated, and embedded in Epon resin. Epon-embedded liver tissue specimens were cut into ultrathin sections and assessed by transmission electron microscopy (H-600, Hitachi). Then, the mitochondrial length/width ratio and mean area were assessed by Image-Pro Plus 6.0 software, as previously described [44], ten mitochondria were randomly selected from each image, the length parameter was used to track and measure the length and width of mitochondria, and mitochondrial area was assessed with area parameter.

### 2.12. Relative mtDNA Copy Number Assay

Relative mitochondrial DNA (mtDNA) copy numbers were detected by qPCR. Firstly, total DNA was extracted from liver samples using the DNA tissue kit (Beyotime, China), and 100 ng DNA was prepared for the qPCR assay. Referring to previously described methods [45], the ratio of mtDNA copy number to nuclear DNA copy number can be calculated by comparing ND1 and 16S expression to HK expression.

### 2.13. Statistical Analysis

Statistical analysis was done using SPSS 20.0 (IBM, Armonk, NY, USA) and GraphPad Prism 8.0 (GraphPad Software, San Diego, CA, USA). All data are expressed as the mean ± standard deviation (SD). The data were tested for normality by the Shapiro-Wilk normality test and Kolmogorov–Smirnov test (*P* > 0.05), and the Brown-Forsythe and Bartlett's tests were run to validate the homoscedasticity assumption (*P* > 0.05). Comparisons between multiple groups were measured by one-way ANOVA, followed by Dunnett's multiple comparisons test. *P* < 0.05 was considered statistically significant.

## 3. Results

### 3.1. Mito-TEMPO Pretreatment Protects Mice against LPS-Induced Acute Liver Injury

**Figure 1:**
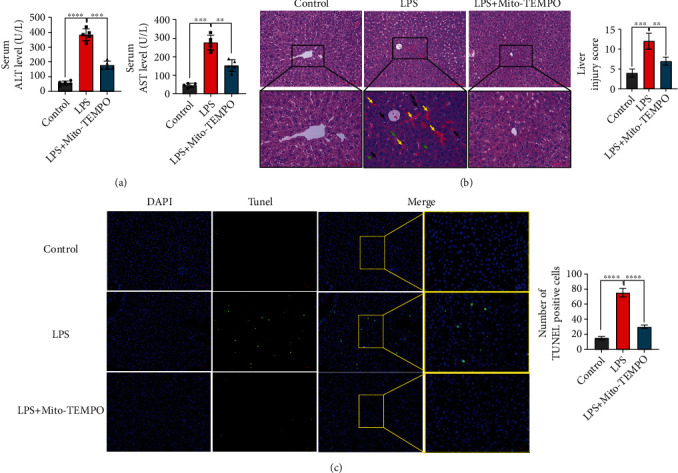
Mito-TEMPO mitigates liver injury in LPS-induced sepsis mice. (a) Serum ALT and AST levels of LPS-induced sepsis mice. (b) Scoring and representative pictures of hepatic H&E staining (scale bar = 100 *μ*m, 50 *μ*m). Liver injury scores were estimated by the following six indicators: vacuolization, nuclear condensation, nuclear fragmentation, nuclear fading, erythrocyte stasis, and inflammatory cell infiltration. Scores were assigned based on the percentage of cells showing these phenomena in five microscopic fields (×200) as follows: 0 = 0%, 1 = 0–10%, 2 = 10–50%, and 3 = 50–100%. The six subscores were summed; the higher the total score, the more severe the injury. (c) The TUNEL-positive cells in the liver of LPS-induced sepsis mice (scale bar = 50 *μ*m, 20 *μ*m). The proportion of positive cells was assessed in five random fields (×200). *n* = 5 per group. Data are presented as the mean ± SD from at least three independent experiments. ^∗^*P* < 0.05, ^∗∗^*P* < 0.01, ^∗∗∗^*P* < 0.005, and ^∗∗∗∗^*P* < 0.0001.

Serum ALT and AST levels are the most used biochemical markers of liver injury. Compared with the control group, the serum levels of ALT and AST were elevated approximately 3- and 4-fold in the LPS group (*P* < 0.0001, Figure 1(a)). In the LPS+Mito-TEMPO group, the levels of ALT and AST were approximately 1.5- and 2-fold lower compared with those in the LPS group (*P* < 0.005, Figure 1(a)). Based on liver histopathology analysis, we found that the liver injury score in the LPS group was markedly increased compared with that in the control group (*P* < 0.005, Figure 1(b)), due to severe congestion (indicated by yellow arrows), nuclear fading (indicated by green arrows), vacuolization, and inflammatory cell infiltration (indicated by black arrows) (Figure 1(b)). However, the observed liver injury was ameliorated by pretreatment with Mito-TEMPO (Figure 1(b)). Similarly, the liver injury score in the LPS+Mito-TEMPO group was decreased compared with that in the LPS group (*P* < 0.01, Figure 1(b)). Furthermore, we found that the number of TUNEL-positive cells in the mouse liver was higher in the LPS group compared with the control group (*P* < 0.0001, Figure 1(c)). However, Mito-TEMPO pretreatment reduced these numbers (Figure 1(c)).

### 3.2. Mito-TEMPO Pretreatment Reduces LPS-Induced Liver Inflammation

**Figure 2:**
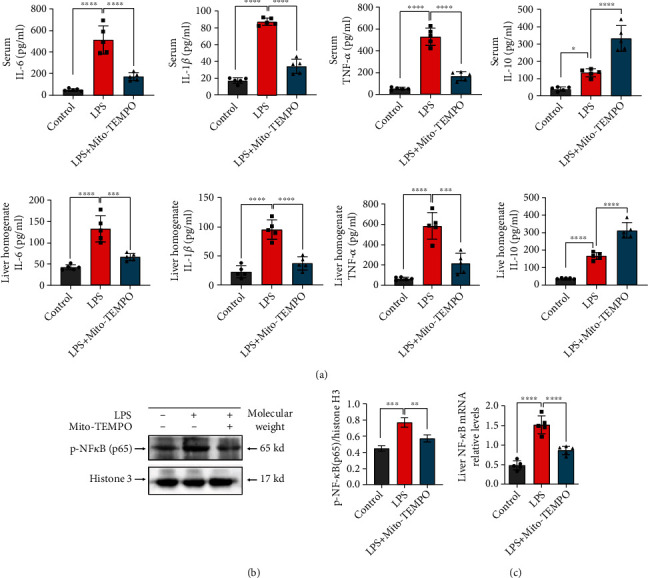
Mito-TEMPO diminishes liver inflammation in LPS-induced sepsis. (a) Expression of cytokines in serum and liver tissue homogenate. (b) Nuclear proteins from mouse liver tissues were extracted, and p-NF-*κ*B (p65) protein levels in the liver were detected by western blotting. (c) NF-*κ*B relative mRNA levels in the liver. *n* = 5 per group. Data are presented as the mean ± SD from at least three independent experiments. ^∗^*P* < 0.05, ^∗∗^*P* < 0.01, ^∗∗∗^*P* < 0.005, and ^∗∗∗∗^*P* < 0.0001.

Inflammation is one of the main factors of liver injury during sepsis [2]. Previous studies indicated that LPS-induced liver inflammation may be mediated by the activation of NF-*κ*B, which increases the transcription of proinflammatory cytokines [46]. In the present study, the qPCR data indicate that the liver mRNA levels of NF-*κ*B were increased after LPS injection compared with the control group (*P* < 0.0001, Figure 2(c)) and decreased after Mito-TEMPO pretreatment compared with the LPS group (Figure 2(c)). To determine the effect of Mito-TEMPO on nuclear translocation of NF-*κ*B (p65), nuclear proteins were extracted from mouse livers for western blotting. We found that Mito-TEMPO pretreatment suppressed LPS-induced NF-*κ*B (p65) nuclear translocation compared with the LPS group (*P* < 0.01, Figure 2(b)). Additionally, we measured the concentrations of cytokines in liver tissue homogenate and serum. LPS injection increased the expression of IL-6, IL-1*β*, and TNF-*α* in the serum and liver homogenate, and Mito-TEMPO pretreatment significantly reduced the levels of these proinflammatory cytokines (Figure 2(a)). The levels of IL-10 slightly increased following LPS challenge and remarkably increased after Mito-TEMPO pretreatment compared with the LPS group (*P* < 0.0001, Figure 2(a)).

### 3.3. Mito-TEMPO Improves LPS-Induced Oxidative Stress in the Liver

**Figure 3:**
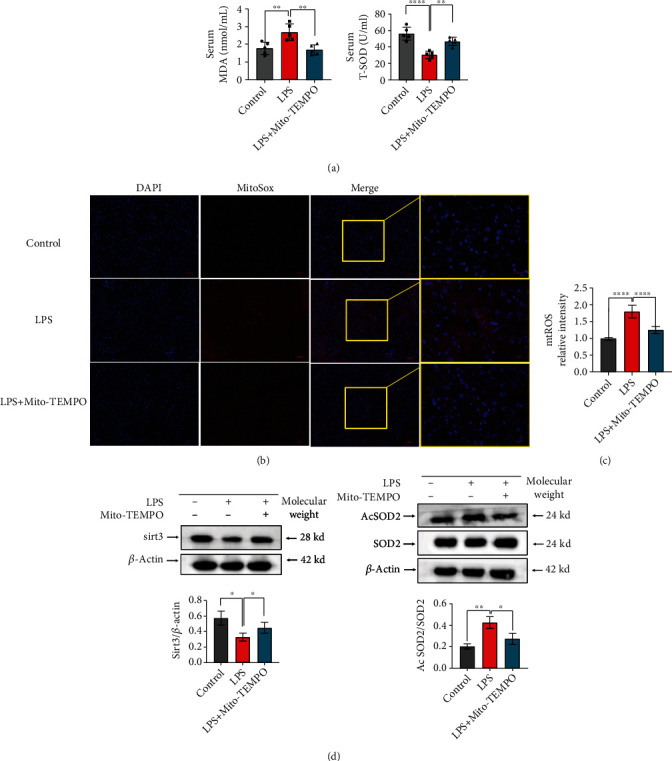
Mito-TEMPO alleviates oxidative stress in LPS-induced sepsis. (a) MDA and SOD activity in serum from LPS-induced sepsis mice. (b, c) Measurement of mitochondrial superoxide production in the liver from LPS-induced sepsis mice using a fluorescent MitoSOX probe (scale bar = 50 *μ*m, 20 *μ*m). (d) The protein expression levels of Sirt3, SOD2, and Ac-SOD2 in the liver were detected by western blot. *n* = 5 per group. Data are presented as the mean ± SD from at least three independent experiments. ^∗^*P* < 0.05, ^∗∗^*P* < 0.01, ^∗∗∗^*P* < 0.005, and ^∗∗∗∗^*P* < 0.0001.

An increasing body of evidence suggests that oxidative stress plays an important role in the pathogenesis of septic liver injury [47, 48]. To evaluate oxidative stress conditions, we measured the antioxidant enzyme activity of SOD and the serum levels of the lipid peroxidation product MDA. Our results show that compared with the LPS group, pretreatment with Mito-TEMPO significantly decreased serum MDA contents (*P* < 0.01, Figure 3(a)) and increased SOD activity (*P* < 0.01, Figure 3(a)). These findings suggest that Mito-TEMPO administration enhanced the antioxidative capability in septic mice, which reflects the ability to neutralize ROS. We measured ROS levels by fluorescence assays; the fluorescence intensity of MitoSOX was elevated in the livers of the LPS group compared with the control group (*P* < 0.0001, Figures 3(b) and 3(c)), but decreased by pretreatment with Mito-TEMPO (Figures 3(b) and 3(c)). We detected the levels of Sirt3 and Ac-SOD2 in the liver using western blot. We found that the levels of Sirt3 were reduced in the LPS group (*P* < 0.05, Figure 3(d)) and that SOD2 acetylation was increased in the LPS group (*P* < 0.01, Figure 3(d)) compared with the control group. However, these results were reversed by Mito-TEMPO pretreatment, showing the potent antioxidative capacity of Mito-TEMPO in vivo.

### 3.4. Mito-TEMPO Attenuates LPS-Induced Mitochondrial Dysfunction

**Figure 4:**
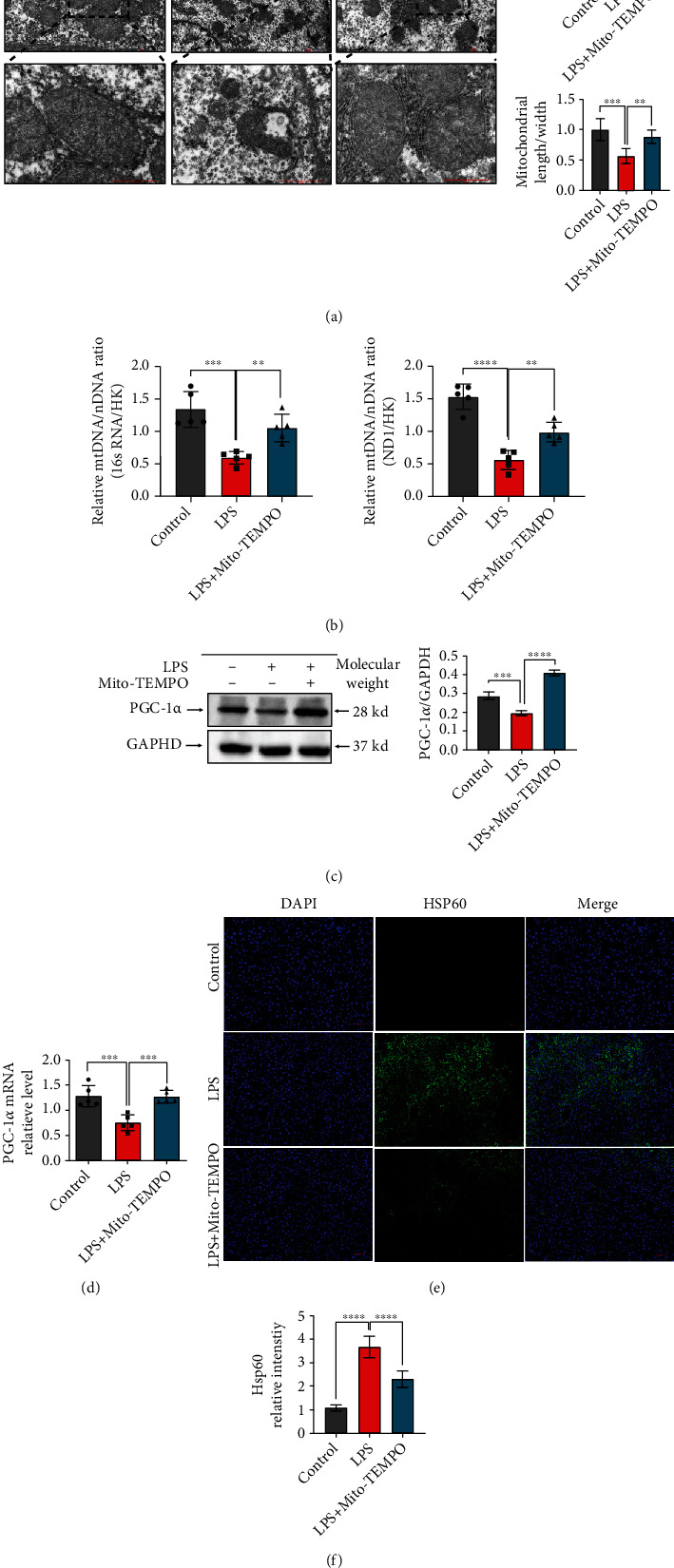
Mito-TEMPO improves mitochondrial function in LPS-induced sepsis. (a) Representative transmission electron microscopy images of mitochondria in the liver of LPS-induced sepsis mice (scale bar = 2 *μ*m, 1 *μ*m). (b) Measurement of mtDNA copy numbers in the liver of mice. (c) The liver protein levels of PGC-1*α* as assessed by western blot. (d) The liver mRNA levels of PGC-1*α* as assessed by qPCR. (e, f) The proportion of HSP60-positive cells in the liver was determined by immunofluorescence (scale bar = 50 *μ*m). *n* = 5 per group. Data are presented as the mean ± SD from at least three independent experiments. ^∗^*P* < 0.05, ^∗∗^*P* < 0.01, ^∗∗∗^*P* < 0.005, and ^∗∗∗∗^*P* < 0.0001.

To confirm the effect of Mito-TEMPO on mitochondrial function, we measured the levels of mitochondrial functional indicators. Mitochondrial morphology was examined by electron microscopy. As shown in Figure 4(a), in the livers of the LPS group, mitochondria were swollen and broken, membrane integrity was disrupted, and mitochondrial cristae were broken or missing (Figure 4(a)). HSP60 is regarded as an indicator of mitochondrial stress, and its levels increase when mitochondria are injured [49]. We found increased HSP60 expression in the LPS group compared with the control group (*P* < 0.0001, Figure 4(e)), but HSP60 expression was decreased by Mito-TEMPO pretreatment (Figure 4(e)). Moreover, the livers of LPS mice showed severe mitochondrial injury, as evidenced by decreased relative mtDNA levels (Figure 4(b)), PGC-1*α* protein (*P* < 0.001, Figure 4(c)) and mRNA levels (*P* < 0.001, Figure 4(d)), mitochondrial area (*P* < 0.001, Figure 4(a)), and mitochondrial length/width ratio (*P* < 0.001, Figure 4(a)) compared to the control group. However, mitochondrial size and function were partially restored in the LPS+Mito-TEMPO group compared with the LPS group.

### 3.5. Mito-TEMPO Relieves Caspase-1 Activation and Pyroptosis in the Liver

**Figure 5:**
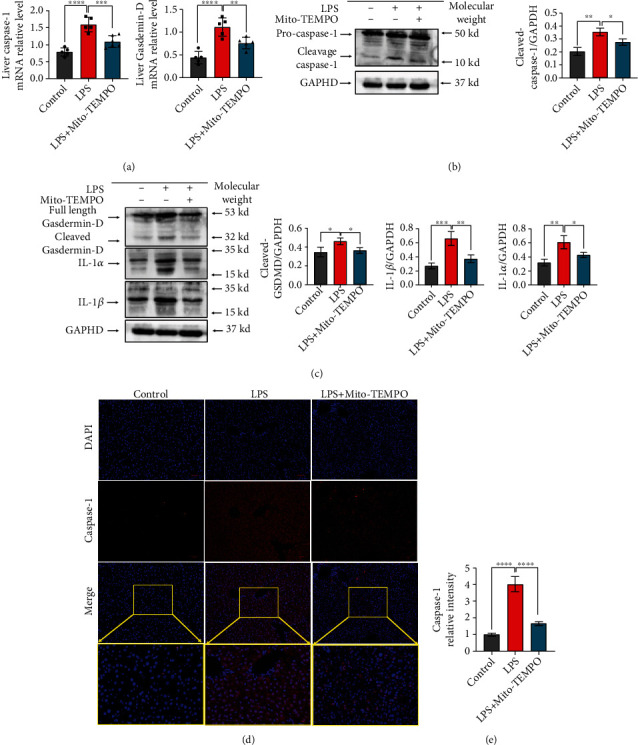
Mito-TEMPO alleviates caspase-1-dependent pyroptosis in the liver upon LPS-induced sepsis. (a) The mRNA levels of caspase-1 and Gasdermin-D. (b, c) The levels of pyroptosis-related proteins as detected by western blot. (d, e) Immunofluorescence assays of caspase-1 (scale bar = 50 *μ*m, 20 *μ*m). *n* = 5 per group. Data are presented as the mean ± SD from at least three independent experiments. ^∗^*P* < 0.05, ^∗∗^*P* < 0.01, ^∗∗∗^*P* < 0.005, and ^∗∗∗∗^*P* < 0.0001.

Sepsis is closely associated with pyroptosis, which is a novel type of programmed cell death [50]. To clarify whether Mito-TEMPO alleviates sepsis-induced liver damage by regulating caspase-1 expression and pyroptosis, we detected the protein and mRNA levels of pyroptosis-related proteins in the liver. Compared with the control group, we found that mRNA levels of caspase-1 (*P* < 0.0001, Figure 5(a)) and GSDMD (*P* < 0.0001, Figure 5(a)) were increased in the LPS group, and Mito-TEMPO pretreatment reduced this effect (Figure 5(a)). Western blot analysis indicated similar results; Mito-TEMPO pretreatment inhibited the expression of cleaved caspase-1, cleaved GSDMD, IL-1*α*, and IL-1*β* (Figures 5(b) and 5(c)). In addition, immunofluorescence showed that the expression levels of caspase-1 were increased in the LPS group compared with the control group (*P* < 0.001, Figure 5(e)), whereas Mito-TEMPO pretreatment reduced caspase-1 expression (Figures 5(d) and 5(e)).

## 4. Discussion

Although inflammation is an essential biological defense mechanism, aberrant immune responses can lead to host tissue damage [51]. Sepsis is characterized by hyperactivation of the immune response, which triggers an excessive inflammatory response. The liver plays a major role in the immune response and releases a large number of inflammatory cytokines. It is also vulnerable to inflammatory injury. Sepsis-related liver damage is an independent risk factor for multiple organ dysfunction and sepsis-induced mortality [3]. The development of new treatment strategies or optimization of existing therapies is important for recovering liver function and improving mortality rates in patients with sepsis. Recently, some studies reported that agents with antioxidative capacity could effectively suppress the systemic immune response and improve the outcome of patients with sepsis. In fact, there are several small molecules that prevent the uncontrolled production of ROS and are known to be beneficial in the maintenance of tissue homeostasis during sepsis [52], such as glucocorticoids [53], Stefin B (an endogenous cysteine cathepsin inhibitor) [54], and maresin 1 (a metabolite of theomega-3 fatty acid) [55]. Targeted delivery of antioxidants to mitochondria has been suggested as a potential therapeutic strategy against sepsis. Mito-TEMPO is a mitochondria-targeted superoxide mimetic that has protective effects against acetaminophen-induced acute liver injury [29]. Recent studies have shown that Mito-TEMPO recovered renal function and improved survival in septic mice [56]. Other results have shown that Mito-TEMPO therapy rescues mice from doxorubicin-induced cardiotoxicity by improving mitochondrial function [57]. In the present study, we found that liver injuries, as indicated by increased ALT and AST activities in serum, were significantly decreased following pretreatment with Mito-TEMPO (Figure 1(a)). In line with this, compared with the LPS group, the LPS+Mito-TEMPO group showed an improvement in the severity of liver injury (Figure 1(b)) and a decrease in the apoptosis of liver cells (Figure 1(c)). The inflammatory response is an initial feature of sepsis. Proinflammatory cytokines in serum are important pathological factors and valuable indicators of sepsis. We observed that the expression levels of proinflammatory cytokines including IL-6, IL-1*β*, and TNF-*α* in serum and liver tissue homogenates of mice were reduced after Mito-TEMPO pretreatment (Figure 2(a)). This may be associated with inhibited nuclear translocation of NF-*κ*B (p65) (Figures 2(b) and 2(c)), which is an important factor upstream of the inflammatory response. Taken together, these results indicate that Mito-TEMPO is able to relieve LPS-induced liver injury and suppress the expression of proinflammatory factors.

Oxidative stress is a common concept in disease [58]. Accumulating evidence has indicated that oxidative stress plays an important role in the pathogenesis of liver injury. One study on hepatitis C virus (HCV) infection suggested that HCV and its proteins/components trigger oxidative stress and inflammation signaling cascades and in turn stimulate ROS production. ROS can lead to host genetic mutation and inflammation, consequently causing liver injury [59]. In the progression of sepsis, the generation of ROS is closely linked to the occurrence and development of liver damage, not only via the direct damage to hepatocytes by promoting the formation of oxidative protein adducts and lipid peroxides but also through the proinflammatory response accentuated by oxidative stress [7]. In fact, as the main metabolic organ responsible for deoxygenation, lipid synthesis, and glycogen storage, the liver is more vulnerable to oxidative stress induced by a variety of factors, including sepsis-associated inflammatory responses [60]. Redox imbalance could cause impairment of biochemical and metabolic processes in hepatocytes. Thus, oxidative stress relief has been suggested as a potential therapeutic strategy against liver injury in sepsis [61]. Oxidative stress results from ROS accumulation or the impairment of ROS clearance and/or oxidative damage repair ability [62]. Studies have shown that impairment of antioxidant capacity leads to increased ROS production [63], and endotoxemia, sepsis, and septic shock are associated with the generation of ROS [64]. Therefore, we studied whether Mito-TEMPO exerted its therapeutic effect on septic liver injury via the prevention of oxidative stress. MDA is an end product of lipid peroxidation, and increased MDA levels reflect oxidative stress [65]. SOD is known to be part of the primary defense system against oxidative stress [66]. In this study, the activity of SOD in serum was increased by pretreatment with Mito-TEMPO; in contrast, Mito-TEMPO pretreatment decreased MDA levels (Figure 3(a)). Furthermore, we found the fluorescence intensity of MitoSOX was decreased by pretreatment with Mito-TEMPO (Figure 3(b)). These results indicate the potent antioxidative capacity of Mito-TEMPO in vivo. Mitochondria are the major organelle for ROS production, and mitochondrial SOD2 is regarded as the main ROS-depleting antioxidant enzyme [67]. A previous study suggested that SOD2 activity was influenced by acetylation at several conserved lysine residues, and Sirt3 plays a major role in maintaining the deacetylation and activity of SOD2, which results in ROS depletion and reduces excessive oxidative stress [68]. Specifically, Sirt3 regulates the expression of SOD2 and exerts its protective effects against ROS and mitochondrial oxidative stress by transforming Ac-SOD2 into SOD2 [69]. One study found that diminished Sirt3 expression significantly increased mtROS production due to SOD2 acetylation, which promotes the development of hypertension [68]. Of note, the Sirt3–SOD2 pathway may play a vital role in modulating mtROS formation. In the present study, we observed that Mito-TEMPO increased Sirt3 levels and decreased Ac-SOD2 levels (Figure 3(d)). These results were consistent with other studies and support the specific effects of mitochondria-targeted drugs on the Sirt3–SOD2 pathway [42].

The mitochondrion is an important organelle for energy generation, calcium homeostasis, ROS production, and cell death [70]. An increasing number of studies have found that mitochondrial injury is the primary pathogenesis site for ischemic disease, cardiomyopathy, and sepsis-related organ failure [71]. Mitochondrial dysfunction can result in cellular and tissue dysfunction and promote perpetuation of infection. Mitochondrial dysfunction is associated with mitochondrial morphological changes, elevated mtROS levels, and mtDNA depletion [72, 73]. In this study, we found evidence of mitochondrial damage reduction in the livers after pretreatment with Mito-TEMPO, which reduced mtROS levels (Figure 3(b)) and partially recovered the liver mtDNA level (Figure 4(b)) and the mitochondrial length/width ratio (Figure 4(a)). HSP60 is normally localized in the mitochondria and is involved in protein folding [74]. However, under stress conditions, HSP60 is translocated to the plasma membrane and released extracellularly. HSP60 is thought to be an indicator of mitochondrial stress [49]. PGC-1*α* is a transcriptional coactivator that acts as a master regulator of mitochondrial biogenesis, mitochondrial respiration, and antioxidant activity. Mitochondrial biogenesis is essential to cell division, and PGC-1*α* promotes mitochondrial biogenesis and oxidative metabolism [75]. In this study, PGC-1*α* and HSP60 levels were partially recovered (Figures 4(c)–4(f)). This further supports the notion that mitochondrial dysfunction is a contributing factor in the induction of liver injury in sepsis. Furthermore, our results also demonstrate that Mito-TEMPO attenuates sepsis-induced liver mitochondrial dysfunction. In addition, mitochondria are recognized to be key regulators of cell death [76]. Previous studies reported that mitochondrial dysfunction leads to the release of proapoptotic proteins, such as cytochrome *c*, resulting in apoptosis, playing a major role in the inflammatory response of sepsis-induced liver injury [77]. Emerging studies have suggested that the mortality of sepsis is largely associated with the development of pyroptosis [78], a new kind of programmed cell death induced by inflammatory caspases [79], resulting not only in cell death but also in excessive inflammatory damage. Pyroptosis can be induced by caspase-1. Active caspase-1 cleaves the pore-forming protein GSDMD, which subsequently forms membrane pores, causing proinflammatory factors release [80]. Research indicated that uncontrolled ROS production from impaired mitochondria is the main trigger of caspase-1 activation and induces pyroptosis [76]. Our results revealed that pretreatment with Mito-TEMPO reduced the expression levels of cleaved caspase-1, cleaved GSDMD, IL-1*α*, and IL-1*β* (Figures 5(b) and 5(c)). These results provide evidence that Mito-TEMPO can mitigate caspase-1 activation and pyroptosis by alleviating cellular oxidative stress and restoring mitochondrial function. Therefore, ameliorating mitochondrial damage and inhibiting the level of ROS may be a viable strategy to suppress pyroptosis and prevent sepsis-associated liver injury.

In summary, sepsis can induce mitochondrial damage, which can cause oxidative stress by the production of ROS and trigger pyroptosis and subsequent cell death. Mito-TEMPO mitigates oxidative stress and alleviates caspase-1-dependent pyroptosis.

## 5. Conclusion

Mito-TEMPO is able to relieve LPS-induced liver injury and suppress the expression of proinflammatory factors. This action of Mito-TEMPO is achieved through the alleviated oxidative stress and restored mitochondrial function. Our results also revealed that ROS exacerbates pyroptosis activation in LPS-induced liver injury and that Mito-TEMPO scavenging of ROS has a protective effect on the septic liver. These findings suggest that targeted delivery of antioxidants to mitochondria may be a potential therapeutic strategy against sepsis.

## Data Availability

The data used to support the findings of this study are available from the corresponding author upon request.
